# First-Principles Prediction of New 2D *p*-SiPN: A Wide Bandgap Semiconductor

**DOI:** 10.3390/nano12224068

**Published:** 2022-11-18

**Authors:** Shambhu Bhandari Sharma, Issam Qattan, Santosh KC, Sufian Abedrabbo

**Affiliations:** 1 Department of Physics, Khalifa University of Science and Technology, Abu Dhabi P.O. Box 127788, United Arab Emirates; 2 Chemical and Materials Engineering, San Jose State University, San Jose, CA 95112, USA

**Keywords:** ab-initio, density functional theory, strain modulation, elastic properties, electronic properties, optical properties

## Abstract

Pentagonal two-dimensional ternary sheets are an emerging class of materials because of their novel characteristic and wide range of applications. In this work, we use first-principles density functional theory (DFT) calculations to identify a new pentagonal SiPN, *p*-SiPN, which is geometrically, thermodynamically, dynamically, and mechanically stable, and has promising experimental potential. The new *p*-SiPN shows an indirect bandgap semiconducting behavior that is highly tunable with applied equ-biaxial strain. It is mechanically isotropic, along the x-y in-plane direction, and is a soft material possessing high elasticity and ultimate strain. In addition, its exceptional anisotropic optical response with strong UV light absorbance, and small reflectivity and electron energy loss make it a potential material for optoelectronics and nanomechanics.

## 1. Introduction

Inspired by the discovery of pentagraphene [1,2], two-dimensional (2D) Cairo-pentagonal lattice-based materials are attracting much attention and emerging as a new class of materials because of their exceptional physiochemical properties, identified mostly with theoretical and computational approaches [3]. Nevertheless, the experimental synthesis of the penta-PbSe${}_{2}$ [4,5], penta-PdS${}_{2}$ [6,7], penta-NiN${}_{2}$ [8] and penta-silecene nanoribbon [9] already sheds light on the experimental feasibility of these compounds. The presence of unique buckling/puckkering with three virtual layers and non-centrosymmetric geometry allow them to demonstrate extraordinary mechanical, piezoelectric, magnetic, electronic, lattice thermal conductivity, and optoelectronic properties, beyond their hexagonal counterpart [3].

More specifically, the light-weight penta compounds comprised of elements from period I-II of the periodic table have gained much interest lately because of their light-mass, high abundance, and environmental characteristics. In addition to the elementary penta-graphene and penta-silicene, the binary pentagonal monolayers including penta-CN${}_{2}$ [10,11,12], penta-SiC${}_{2}$ [13,14], penta-SiN${}_{2}$ [15], penta-Si${}_{5}$C [16], penta-BN${}_{2}$ [17], penta-B${}_{2}$C [18], penta-P${}_{2}$C [19], and BP${}_{5}$ [20] demonstrate multi-functional responses and properties ranging from outstanding mobility, thermal conductivity, to electronic and transport behavior suitable for many applications such as thermoelectricity, photo- and electro-catalysis, and heterostructures devices.

Moving one step beyond the binary, recent predictions of ternary penta compounds such as penta-BCN [21], penta-CNP [22], penta-BNSi [23], penta-SiCN [24], penta-BCP [25], and penta-BPN [26] have shown high chemical, mechanical, thermodynamical and kinetic stability and prominent piezoelectricity, photocatalytic activity and auxetic mechanics, that are even superior to that of binary composition. The breaking of centrosymmetry with a larger number of ingredients provides a higher degree of freedom to ternary compounds allowing them to inherit exceptional properties.

A large number of new, undiscovered penta 2D materials are waiting to be revealed, and in particular, the ternary penta-sheets are currently of high research interest. The quest for new stable and experimentally feasible novel multi-functional materials is crucial to fulfilling the demand for futuristic cutting-edge technological applications.

It is critical to emphasize the fact that favorness of N, P, and Si in buckled configuration and flexible formation of the electron cloud of P atom as well as the comparable bond lengths and bond angles support genuine mechanical strength to form a new pentagonal compound. Considering all these factors together, we design a new pentagonal silicon phosphorus nitride (*p*-SiPN) compound, which is structurally, dynamically, thermodynamically, and mechanically stable.

We utilize first-principles density functional theory (DFT) calculations and theoretical analysis to identify the structural, chemical, thermodynamical, mechanical, and dynamical stability of the new *p*-SiPN monolayer, and predict the possibility of future experimental synthesis. Further, the unique properties of *p*-SiPN including its geometry, tunable semiconducting behavior, and outstanding elastic properties including the in-plane stiffness or the 2D-Young’s modulus, Poisson’s ratio, shear modulus, the ultimate strength, as well as critical strain, and their mechanical stability criterion are thoroughly explored. Additionally, the presence of mechanical isotropy, high elasticity, and strength of *p*-SiPN are intriguing in nanomechanics. Furthermore, the impressive optical properties such as anisotropic and intense optical absorbance, average static dielectric constants and refractive index, and reflectivity show *p*-SiPN to be an important ternary compound of the pentagonal family with potential applications in optoelectronics and nanomechanics relevant to both the scientific and industrial communities.

## 2. Computational Details

The Spanish Initiative for Electronic Simulations with Thousands of Atoms (SIESTA) [27,28] code is utilized to perform all DFT calculations. The generalized gradient approximation (GGA)—Perdew-Burke-Ernzerhof (PBE) [29] parametrization is used to account for both the electronic exchange and correlation potentials. In addition, the Troullier-Martins [30] norm-conserving pseudopotentials within the semi-local form are utilized to estimate the electrons-core interactions based on the Kleinman-Bylander factorized form. The double-ζ polarized (DZP) basis set assembled from numerical atomic orbitals (NAO) of the finite range is used to describe the electronic distribution of each atom. A 20 × 20 × 1 k-points mesh under the Monkhrost pack scheme [31], and 350 Ry of the cutoff energy, establish convergence criterion. Atomic forces lower than 0.01 eV/Å, and 10${}^{-6}$ eV self-consistent field (SCF) integrated in the conjugate-gradient (CG), are set for geometry optimization. Furthermore, to remove the spurious interactions between adjacent unit cells, we create a thick vacuum-gap of 25 Å along the z-direction (out-of-plane). To ensure the chemical stability, both cohesive ($E_{coh}$), and formation ($E_{f}$) energies [25] are evaluated:

(1) $$\begin{array}{c} E_{coh}=\left(E_{p-SiPN}-2E_{Si}-2E_{P}-2E_{N}\right)/6 \end{array}$$

where $E_{p-SiPN}$, $E_{Si}$, $E_{P}$, and $E_{N}$ denote the energy of *p*-SiPN, free/isolated Si, P, and N atoms, respectively. Similarly,

(2) $$\begin{array}{c} E_{f}=\left(E_{p-SiPN}-2E_{P}^{Bulk}-2E_{Si}^{Bulk}-2E_{N}^{Bulk}\right)/6 \end{array}$$

where $E_{P}^{Bulk}$, $E_{Si}^{Bulk}$, and $E_{N}^{Bulk}$ are the energy of P, Si, and N atoms at their most stable bulk geometry, respectively. To investigate the dynamical stability of *p*-SiPN, we calculate the phonon bands based on the finite displacement method built-in the PHONOPY package [32] as integrated in the Viena Ab-initio Simulation Package (VASP) [33]. For thermodynamical stability of *p*-SiPN, we perform ab-initio molecular dynamics (AIMD) simulations at temperature *T* = 300 K, up to 2000 fs in a time-step of 1 fs. For both dynamical and thermodynamical calculations, a 7×7×1 supercell to achieve convergence.

First–order time–dependent perturbation theory (TDP) [34] within the random phase approximation (RPA) [35,36,37] implemented in SIESTA is used to perform the optical properties. A 60×60×1 k-mesh is chosen, and an optical broadening of 0.1 eV is used to achieve convergence. Optical properties in the light-energy range (0–10) eV, covering all regions: infrared (IR), visible (VIS), and ultraviolet (UV) regions, and for both parallel (in-plane), and perpendicular (out-of-plane) directions are investigated.

In general, the dielectric function ε(ω) is expressed as:

(3) $$\varepsilon \left(\omega \right)=\varepsilon_{1}\left(\omega \right)+i\varepsilon_{2}\left(\omega \right),$$

with $\varepsilon_{1}\left(\omega \right)$/$\varepsilon_{2}\left(\omega \right)$ being the real/imaginary part of ε(ω). The real part $\varepsilon_{1}\left(\omega \right)$ is calculated based on the Kramer-Kronig transformation (KK) [38] of $\varepsilon_{2}\left(\omega \right)$:

(4) $$\varepsilon_{1}\left(\omega \right)=1+\frac{2}{\pi }P\int_{0}^{\infty }\frac{\varepsilon_{2}\left(\omega^{\prime }\right)\omega^{\prime }}{\omega^{\prime^{2}}-\omega^{2}}d\omega^{\prime },$$

where *P* is the principle part of $\varepsilon_{1}\left(\omega \right)$ [39]. For $\varepsilon_{2}\left(\omega \right)$, TDP is used for the calculations:

(5) $$\begin{array}{c} \varepsilon_{2}\left(\omega \right)=\frac{e^{2}}{\omega^{2}\pi m^{2}}\sum_{v,c}\int_{BZ}d\vec{k}{\left|\langle \psi_{ck}\left|\hat{e}\cdot \vec{p}\right|\psi_{vk}\rangle \right|}^{2}\\ \delta \left(E_{c}\left(k\right)-E_{v}\left(k\right)-\hbar \omega \right)), \end{array}$$

with a clear electronic-optical coupling. In Equation (5) above, v/c refers to the valence/conduction band states, whereas $E_{\left(c,v\right)}\left(k\right)$/$\psi_{(c,v),k}$ is the energy/eigen function of the $k_{th}$-state, respectively. Here, the momentum-operator and polarization-vector are denoted by $\vec{p}$ and $\hat{e}$, respectively.

The extinction coefficient K(ω) and the refractive index η(ω) are calculated using:

(6) $$K\left(\omega \right)={\left(\frac{\sqrt{\varepsilon_{1}^{2}\left(\omega \right)+\varepsilon_{2}^{2}\left(\omega \right)}-\varepsilon_{1}\left(\omega \right)}{2}\right)}^{\frac{1}{2}},$$

and

(7) $$\eta \left(\omega \right)={\left(\frac{\sqrt{\varepsilon_{1}^{2}\left(\omega \right)+\varepsilon_{2}^{2}\left(\omega \right)}+\varepsilon_{1}\left(\omega \right)}{2}\right)}^{\frac{1}{2}},$$

and then K(ω) is used to calculate the reflectivity R(ω), electron energy-loss function *L*(ω), and absorption coefficient α(ω) as:

(8) $$R\left(\omega \right)=\frac{{\left[1-n\left(\omega \right)\right]}^{2}+K^{2}\left(\omega \right)}{{\left[1+n\left(\omega \right)\right]}^{2}+K^{2}\left(\omega \right)},$$

(9) $$L\left(\omega \right)=\frac{\varepsilon_{2}^{2}\left(\omega \right)}{\varepsilon_{1}^{2}\left(\omega \right)+\varepsilon_{2}^{2}\left(\omega \right)},$$

and

(10) $$\alpha \left(\omega \right)=\frac{2\omega K(\omega )}{c}.$$

## 3. Results and Discussions

### 3.1. Structural Properties

**Figure 1:**
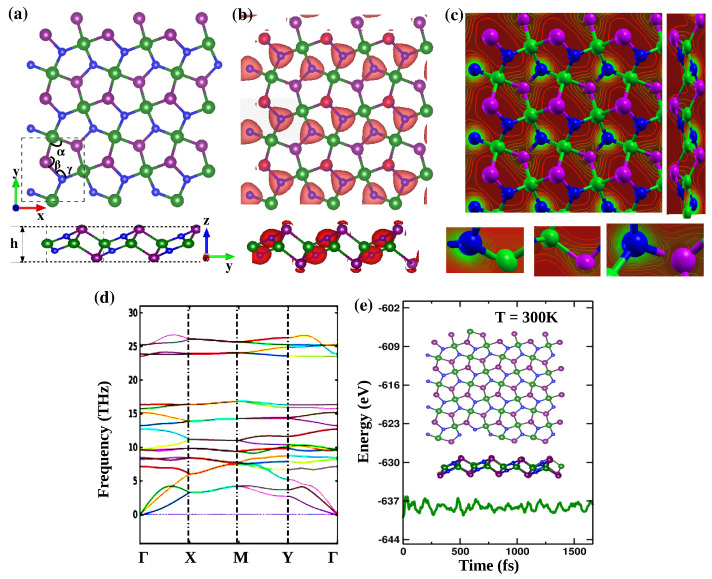
(Color online) (**a**) Top-view (upper figure) and side-view (bottom figure) of the optimized geometry of *p*-SiPN monolayer. The green, purple, and blue balls are a representation of the Si, P, and N atoms, respectively, (**b**) valence charge density iso-surface shown in light-magenta (0.11 e/Å${}^{3}$), (**c**) Top (right panel) and side (left panel) showing 2D charge density contour plots of the interatomic charge distribution (bottom), (**d**) phonon band, and (**e**) AIMD simulation showing the energy fluctuation during the NVT ensemble at *T* = 300 K. The inset depicts the top-view (up) and side-view (down) of the final geometry of *p*-SiPN.

Following the configuration of pentagraphene, which is composed of 3-coordinated and 4-coordinated C atoms in the upper/bottom and middle layer, the geometry of *p*-SiPN is constructed by introducing 4-coordinated Si (Si${}_{4c}$) as central, and 3-coordinated P/N (P/N${}_{4c}$) as upper/bottom layer. Other possible pentagonal structures comprising Si, P, and N atoms are also tested. However, the Si${}_{4c}$ is the most stable geometrically and energetically (Figure 1a). The unit cell of *p*-SiPN consists of 2 atoms of each Si, P, and N atom in equal proportion forming 3 virtual layers composited in a monolayer. The initially designed geometry of *p*-SiPN is completely optimized to maintain the lowest possible energy, inter-atomic forces, and stresses. The final structure inherits the *P-2${}_{1}$* crystal symmetry of the space group No. 4, with 2D pentagonal symmetry. The lattice parameters and atomic coordinates of *p*-SiPN are presented in Supporting Information. The obtained lattice parameters of *p*-SiPN are a = 4.41Å and b = 4.43 Å. Interestingly, the top view of the supercell displays four distinct irregular pentagonal Cairo, composed exclusively of Si, P, and N atoms, which preserves the periodic boundary conditions.

The geometrical measurements of the fully relaxed stable configuration of *p*-SiPN shows that the bond length of Si–P, P–N and Si–N are 2.32 Å, 1.81 Å, and 1.78 Å, respectively. The calculated thickness (*h*) is 2.85 Å. This value is large compared to those obtained for penta- graphene (1.20 Å) [2], penta-SiCN (1.24 Å) [24], penta-BCN (1.34 Å) [21], penta-CNP (2.41 Å) [22], penta-BP${}_{5}$ (2.50 Å) [20], and penta-CN${}_{2}$ (1.52 Å) [10]. The higher thickness is attributed to the relatively larger bond-lengths of *p*-SiPN compared to other pentagonal monolayers. The obtained bond angles of P–Si–P (α), Si–P–N (β), and P–N–Si (γ) are 109.30${}^{\circ }$, 97.21${}^{\circ }$, and 119.67${}^{\circ }$, respectively.

We also have performed Mulliken charge density analysis to get more insight into the atomic charge distribution as well as the bonding mechanism. The valence charge density iso-surface plot (Figure 1b) depicts that the charge is accumulated mostly around the N atom, and highly dispersed between the Si–N, and N–P bonds, which is attributed to the higher electronegativity of the N atom. Furthermore, the 2D contour plot of the charge density (Figure 1c) is analyzed in order to get a better understanding of the bonding mechanism. The maximum and minimum intensity of charge density is depicted in green and red colors, respectively. The existence of a higher electronic charge (shown in green color) between Si–N, and P–N bonds, overlapping of concentric lines, and conjoining of the electronic wavefunctions clearly confirm the covalency, which is similar to that of *p*-SiCN [24]. On the other hand, the distinctive smaller overlapping of wavefunctions and the deforming of the conjoining contour lines (dumbbell-shaped) with relatively smaller charge dispersion (faded-green) between P–N indicate the formation of ionic and covalent bonding. According to the intensity of charge distribution between the atoms, the chemical bonds of P–N and Si–N are the strongest compared to that of Si–P.

To test the average atomic binding strength and geometrical stability, the cohesive energy (*E${}_{c}$*) of *p*-SiPN is calculated (Equation (1)), and found to be −5.28 eV/atom. The reasonable negative *E${}_{c}$* suggests the structural stability of the monolayer. In addition, the formation energy ($E_{f}$) is found to be 1.56 eV/atom (Equation (2)) indicating the possibilities of experimental synthesis of *p*-SiPN via endothermic process [25].

In addition, the presence of only real frequency mode in the phonon band spectrum along the first Brillouin zone verifies the sustainable lattice vibration and dynamical stability of the monolayer (Figure 1d). Since the unit cell of *p*-SiPN comprised of 6 atoms, 15 optical and 3 acoustic branches of phonon frequencies with high vibration are observed, which are also observed in penta graphene [1,2]. We have also performed AIMD simulation at room temperature (*T* = 300 K) (Figure 1e) to verify the thermodynamic response of *p*-SiPN. The total potential energy is consistent with the time period with a constant magnitude. In addition, the structure of the monolayer does not suffer any distortion or transformation, indicating robust thermal stability of *p*-SiPN.

### 3.2. Mechanical Properties

To investigate the mechanical response of *p*-SiPN, the required linear elastic tensors (*C*${}_{ij}$) are calculated based on the strain versus strain-energy method [24]. Following Voigt notation [1,40], we calculate the strain-energy per unit area (*A*) as a function of strain $U_{s}\left(\varepsilon \right)=E_{s}$(ε)/*A* using:

(11) $$U_{s}\left(\varepsilon \right)=\frac{1}{2}C_{11}\varepsilon_{xx}^{2}+\frac{1}{2}C_{22}\varepsilon_{yy}^{2}+C_{12}\varepsilon_{xx}\varepsilon_{yy}+2C_{66}\varepsilon_{xy}^{2},$$

where the tensors $C_{11}$, $C_{22}$, $C_{12}$, and $C_{66}$ are extracted by fitting the strain-energy obtained for the uniaxial ($\varepsilon_{x}$ and $\varepsilon_{y}$), and biaxial ($\varepsilon_{xy}$) strains. The mechanical anisotropy is analyzed following the Li’s elastic anisotropy approach [41]. In addition, the anisotropy indices, which include: the universal *A*${}^{\mathrm{SU}}$, Ranganathan *A*${}^{\mathrm{Ranganathan}}$ [42], and Kube *A*${}^{\mathrm{Kube}\;}$ [43] are also calculated using:

$$A^{\mathrm{SU}}={\left(\begin{array}{c} {\left[\frac{1}{4}\left(C_{11}+C_{22}+2C_{12}\right)\left(S_{11}+S_{22}+2S_{12}\right)-1\right]}^{2}\\ +2{\left[\frac{1}{16}\left(C_{11}+C_{22}-2C_{12}+4C_{66}\right)\left(S_{11}+S_{22}-2S_{12}+S_{66}\right)\right]}^{2} \end{array}\right)}^{\frac{1}{2}},$$

(12) $$A^{\mathrm{Ranganathan}\;}=\frac{K^{V}}{K^{R}}+2\frac{G^{V}}{G^{R}}-3\geq 0,$$

and

(13) $$A^{\mathrm{Kube}\;}=\sqrt{{\left(\ln\frac{K^{V}}{K^{R}}\right)}^{2}+2{\left(\ln\frac{G^{V}}{G^{R}}\right)}^{2}},$$

where *K*${}^{V}$/*K*${}^{R}$, and *G*${}^{V}$/*G*${}^{R}$ represent the area/shear moduli for both the Voigt and Reuss parameters, respectively, and defined as [41]:

(14) $$\begin{array}{cc} K^{V} & =\frac{C_{11}+C_{22}+2C_{12}}{4},\\ G^{V} & =\frac{C_{11}+C_{22}-2C_{12}+4C_{66}}{8},\\ K^{R} & =\frac{1}{S_{11}+S_{22}+2S_{12}},\\ G^{R} & =\frac{2}{S_{11}+S_{22}-2S_{12}+S_{66}}. \end{array}$$

The elements of the compliance matrix *S*${}_{ij}$ in Equation (14), are the reciprocal of the $C_{ij}$ tensors [41]. In addition, the angular dependence of the Poisson’s ratio ν(θ), Young’s modulus Y(θ), and shear G(θ) modulus in the range (0${}^{\circ }$ ≤ θ ≤ 360${}^{\circ }$) are also calculated using [24]:

$$\begin{array}{c} \nu \left(\theta \right)=\frac{-C_{12}\left[\cos^{4}\left(\theta \right)+\sin^{4}\left(\theta \right)\right]+N\cos^{2}\left(\theta \right)\sin^{2}\left(\theta \right)}{C_{22}\cos^{4}\left(\theta \right)+M\cos^{2}\left(\theta \right)\sin^{2}\left(\theta \right)+C_{11}\sin^{4}\left(\theta \right)}, \end{array}$$

$$\begin{array}{c} Y\left(\theta \right)=\frac{\left[C_{11}C_{22}-C_{12}^{2}\right]}{C_{22}\cos^{4}\left(\theta \right)+M\cos^{2}\left(\theta \right)\sin^{2}\left(\theta \right)+C_{11}\sin^{4}\left(\theta \right)} \end{array}$$

and

(15) $$\begin{array}{c} \frac{1}{G(\theta )}=\left[S_{11}+S_{22}-S_{12}\right]\cos^{2}\left(\theta \right)\sin^{2}\left(\theta \right)+\\ \frac{1}{4}S_{66}\left[\cos^{4}\left(\theta \right)+\sin^{4}\left(\theta \right)-2\sin^{2}\left(\theta \right)\cos^{2}\left(\theta \right)\right] \end{array}$$

where $M=\left(C_{11}C_{22}-C_{12}^{2}\right)/C_{66}-2C_{12}$, and $N=C_{11}+C_{22}-\left(C_{11}C_{22}+C_{12}^{2}\right)/C_{66}$.

The values of $C_{11}$, $C_{22}$, $C_{12}$ and $C_{66}$ are determined to be 91.76, 92.98, 3.29, and 44.23 N/m, respectively (Table 1). The the Born-Huang criterion [44] are used to establish the mechanical stability: *C*${}_{11}$*C*${}_{22}$−*C*${}_{11}$${}^{2}$> 0, and $C_{66}$> 0. The mechanical stability suggests that lattice distortion occurs after proactive $E_{s}$(ε). The 2D Young’s modulus (*Y*) and the Poisson’s ratio (ν) are calculated along the x-axis (100) and y-axis (010) directions. The *Y* modulus along the (100) direction *Y*${}_{x}$ = $\left(C_{11}C_{22}-C_{12}C_{21}\right)/C_{22}$, and along the (010) direction *Y*${}_{y}$ = $\left(C_{11}C_{22}-C_{12}C_{21}\right)/C_{11}$ are found to be 91.64 and 92.86 N/m, respectively. The small and close values of $Y_{x/y}$ indicate that *p*-SiPN is a relatively softer and mechanically isotropic material, which is different than other ternary penta monolayers [1,22,23,24] (Table 1). On the other hand, the absolute value of the shear modulus is $G_{xy}$ = 44.23 N/m, which is considerably less than that of penta monolayers [21,22,24] (Table 2). Similarly, the Poisson’s ratio in the respective orientations ($\nu_{x}$=C${}_{12}/$$C_{22}$ and $\nu_{y}$=$C_{12}/$$C_{11}$) are 0.035 and 0.036, respectively, supporting isotropic mechanics.

**Figure 2:**
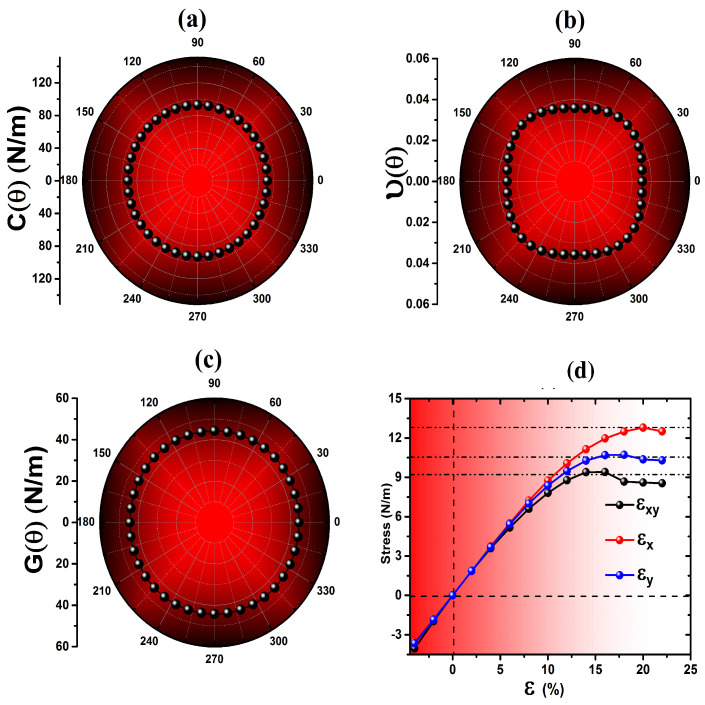
(Color online) The angular dependent of (**a**) 2D Young’s modulus, (**b**) Poisson’s ratio, (**c**) shear modulus, and (**d**) stress-strain energy profile for *p*-SiPN.

The calculated anisotropy indices are listed in Table 2. A value very close to zero indicates an isotropic elastic behavior, while a value larger than zero reveals the scale of anisotropy. The zero indices of $A^{\mathrm{SU}}$, $A^{\mathrm{Ranganathan}\;}$, and $A^{\mathrm{Kube}\;}$ mathematically validate the mechanical isotropy, which is shown by the polar diagram (Figure 2a–c). The angular dependence, shown as polar plots, in the range 0${}^{\circ }$≤ θ ≤ 360${}^{\circ }$ for *Y*(θ) and ν(θ) show perfect circles, suggesting the existence of mechanical isotropy (Figure 2a,b). In contrast, the small distorted circle of *G*(θ) shown in the polar plot suggests a quasi-anisotropic mechanical behavior (Figure 2c).

To investigate the ultimate bond and structure breaking, as well as the elastic-plastic region, high strains uniaxial ($\varepsilon_{x}$ and $\varepsilon_{y}$) and biaxial ($\varepsilon_{xy}$) directions are applied, allowing complete atomic relaxation until *p*-SiPN is completely broken, where deformation is seen. The stress increases linearly with all modes of strain applied, reaching saturation, and then starts to drop (Figure 2d). The stress for all modes of strain applied reaches its maximum value, ultimate stress U${}_{x}$ = 12.79 N/m, U${}_{y}$ = 10.37 N/m, and U${}_{xy}$ = 9.40 N/m, with corresponding critical strain value of $\varepsilon_{xc}$ = 20%, $\varepsilon_{yc}$ = 18%, and $\varepsilon_{xyc}$ = 16%, respectively. The stress clearly shows a parabolic curve at higher strain in the range 8% ≤ $\varepsilon_{x}$ ≤ 20%, indicating a metastable elastic range of *p*-SiPN. Our results show that the ultimate stress, and critical strain of *p*-SiPN are relatively higher than those reported for other penta ternary [22,23,24] monolayers.

### 3.3. Electronic Properties

**Figure 3:**
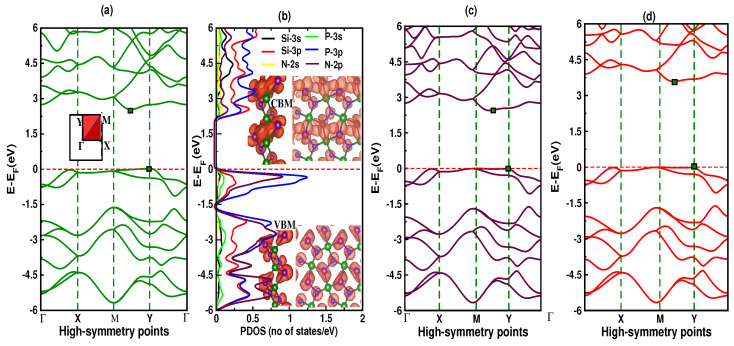
(Color online) (**a**) the electronic band structure, (**b**) partial density of states (PDOS) along with the partial charge densities of the VBM (bottom panels) and CBM (top panels) calculated using GGA-PBE as implemented in SIESTA, (**c**) the band structure obtained using GGA-PBE approximation, and (**d**) the band structure obtained using HSE06 functional as implemented in VASP is also shown for comparison. The green box indicates the VBM and CBM extremity points.

We calculate the spin-polarized/unpolarized electronic bands structure, and partial density of states (PDOS) to gain a comprehensive understanding of electronic behavior of *p*-SiPN. The symmetry observed in both the spin-up and spin-down channels, with a zero total magnetic moment, reveals that *p*-SiPN exhibits non-magnetic ground state. For the electronic band calculation, we choose the high-symmetry points along the Γ–X–M–Y–Γ path of the first Brillouin zone (BZ) using both NAO-basis as implemented in SIESTA (Figure 3a), and the plane-wave approach implemented in VASP (Figure 3c). Both approximations show that the valence-band-maximum (VBM) lies at Y and the conduction-band-minimum (CBM) appears between the M–Y path with a clear gap of 2.43 eV. This identifies *p*-SiPN as an indirect bandgap semiconductor. Interestingly, a unique flat band is observed near the Fermi level of the valence band, which is crucial for quantum materials applications.

Because the standard GGA-PBE approximation underestimates the bandgap in DFT calculations, we have also employed the hybrid functional HSE06 as implemented in VASP for a better quantitative analysis of the bandgap (Figure 3d). Even though the electronic band structure is similar in both approximations, the bandgap value; however, using the HSE06 approximation is found to be 3.43 eV. Furthermore, the PDOS plot (Figure 3b) demonstrates that the P-3*p*, N-2*p*, and Si-3*p* with P-3*s* electronic orbitals ($sp^{3}$-hybridized) share the contribution in descending order to the VBM. On the other hand, the Si-3*p*, P-3*p*, and N-2*p* with N-2*s* contribute significantly to the CBM. The partial charge densities of the VBM (bottom right) and CBM (top right) also demonstrate the similar atomic charge distribution in the VBM and CBM regions. It is worth mentioning here that the asymmetry in the charge distribution in these regions suggests possible piezoelectricity, which is beyond the scope of this work, and yet to be confirmed in a future study.

**Figure 4:**
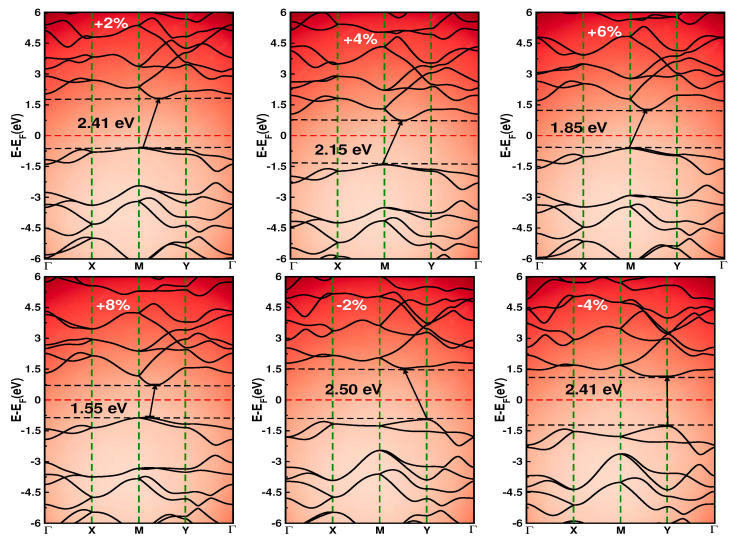
(Color online) The modulation of the electronic band with bi-axial ($\varepsilon_{xy}$) strain. The dashed horizontal lines represent VBM and CBM. The solid arrow represents the nearest inter-band transition.

It is highly desirable to modulate the bandgap of semiconductors for potential electronic switching and sensor applications. Therefore, we load equ-biaxial tensile and compressive strain (−4% ≤$\varepsilon_{xy}$≤ +8%) to investigate the qualitative electronic bandgap tuning mechanism of *p*-SiPN (Figure 4). During the strain loading, the atomic positions are allowed to relax to achieve the minimum energy state. Starting with the compressive strain of $\varepsilon_{xy}$ = −2%, the bandgap increases to 2.50 eV, retaining the indirect nature. However, at $\varepsilon_{xy}$ = −4% the bandgap surprisingly decreases to 2.41 eV with a direct bandgap at the Y point. This anomaly might be due to the meta-stability feature of 2D materials against the compressive strain, as reported previously [45,47].

On the other hand, applying tensile strain at $\varepsilon_{xy}$ = +2%, the indirect bandgap value becomes 2.41 eV suddenly shifting the VMB point from Y to M. For $4\%\leq \varepsilon_{xy}\leq 8\%$, the bandgap is reduced to a value of 2.15 eV, 1.85 eV, and 1.55 eV for $\varepsilon_{xy}$ = 4%, $\varepsilon_{xy}$ = 6%, and $\varepsilon_{xy}$ = 8%, respectively. The reduction in the bandgap value by ≈36% at 8% of stretching, compared to strain-free, reflects a highly tunable electronic response of *p*-SiPN against strain. In general, the reduction (increment) of the bandgap value of *p*-SiPN against tensile (compressive) strain is mainly attributed to the interactions and electronic charge transfer between electronic orbitals. This electronic response against the strain of p-SiPN follows the same trend observed in other ternary penta monolayers [45,47].

### 3.4. Optical Properties

The optical properties are calculated in the range of (0–10) eV for both the in-plane (E||x), and out-of-plane (E||z) directions of light polarization. Overall, the optical activity is clearly enhanced along the E||x direction compared to that of the E||z direction. This is clearly evidenced by the higher intensity seen, and the larger number of spectral peaks obtained.

**Figure 5:**
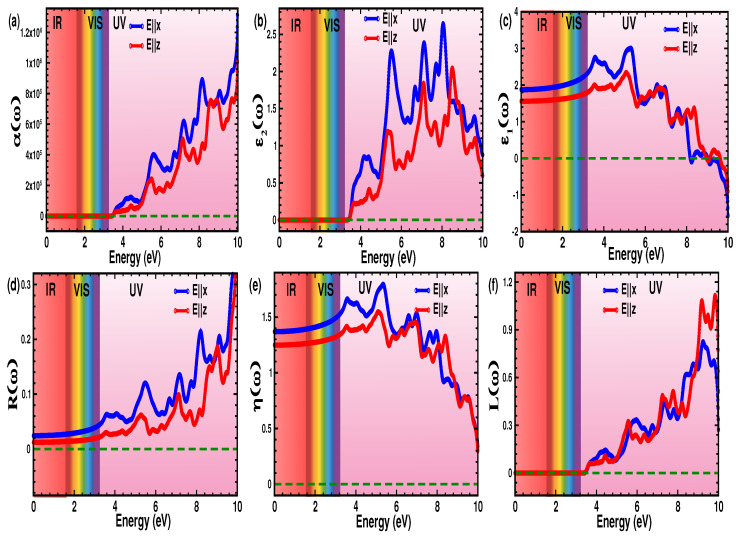
(Color online) (**a**) Absorption coefficient α(ω), (**b**) imaginary part of dielectric function $\varepsilon_{2}\left(\omega \right)$, (**c**) real part of dielectric function $\varepsilon_{1}\left(\omega \right)$, (**d**) reflectance R(ω), (**e**) refractive index η(ω), and (**f**) electron loss function L(ω) as functions of photon energy E(ω). The blue and red solid curves represent the in-plane and out-of-plane direction of incident light polarization, respectively.

The light absorption behavior of a material is attributed to the electronic transitions between the occupied- and unoccupied-states, and is studied by analyzing the absorption coefficient α(ω). The minimum energy needed to excite the absorption spectra, absorption edges ($A_{e}$), is observed to be at 3.43 eV (Figure 5a), which is equivalent to the electronic bandgap value of 3.41 eV. This implies a photo-excited transfer of electrons from the VBM to the CBM. Because of the large bandgap of *p*-SiPN, the absorption peaks are absent in both the IR and VIS regions, which makes *p*-SiPN a superior material for a variety of optical applications such as optical fibers, beam splitters, and UV light operating devices [47,48]. The intensity of the absorption peaks increases with higher light energy (3.43–10.00) eV, with the highest peaks observed at 8.16 eV (≈9.02 × 10${}^{5}$${\mathrm{cm}}^{-1}$), and at 8.61 eV (≈7.63 × 10${}^{5}$${\mathrm{cm}}^{-1}$) for the E||x and the E||z light-directions, respectively. The remarkable wide-range intense absorbance and collective peaks throughout the UV region make *p*-SiPN an outstanding UV light absorbing material. In addition, the enhanced optical absorption and anisotropy add their functionality and importance to light polarizers and waveguides.

To understand the energy-stored as well as the electronic polarizability of the *p*-SiPN monolayer as defined by the Clausius-Mossotti relation [49], we investigate the real part of the dielectric function $\varepsilon_{1}$(ω) for both incidence directions of light (Figure 5b). The optical spectra of $\varepsilon_{1}(\omega$) are consistent from the IR to VIS regions and start to increase from the near UV region followed by a continuous drop, reaching a negative value after 8.53 eV for both light polarizations. The non-negative value of $\varepsilon_{1}\left(\omega \right)$ in the energy range (0–8.53) eV for both light directions suggests the enduring semiconducting activity of *p*-SiPN in that range. Most importantly, the value of $\varepsilon_{1}\left(\omega =0\right)$ is defined as the static dielectric function $\varepsilon_{1}\left(0\right)$, and is found to be 1.88 and 1.55 for the E||x and E||z, respectively (Table 3). Clearly, the $\varepsilon_{1}\left(0\right)$ value for the E||x direction is lower than that of other ternary pentagonal such as *p*-BCN and *p*-SiCN including penta-graphene [50], and other 2D monolayers [51,52].

The interplay between the electronic orbitals, which is responsible for the observed spectral peaks in $\varepsilon_{2}\left(\omega \right)$, justifies the inter-band transitions. These transitions are mostly contributed by the electron alternation of the *p* orbitals of the Si, P, and N atoms (PDOS in Figure 3b). The similar trend of $\varepsilon_{2}\left(\omega \right)$ curve with α(ω) demonstrates their optical coupling as described by Equations (5) and (10). The number and intensity of peaks (Figure 5c) are only observed in the UV region (4.11–9.25) eV, and predominant for the E||x direction compared to the E||z direction, with a clear blue-shift. The higher peaks at 4.15 eV, 5.52 eV, 6.67 eV, 7.13 eV, and 8.10 eV along the E||x direction show the first five inter-band transitions. Overall, the several observed intense peaks in the UV suggest numerous inter-band transitions and intense optical activity.

The reflectivity response R(ω) of *p*-SiPN at different energy ranges (Figure 5d) shows that the reflection is negligible (≈2%) in the IR region, and increases gradually beyond the VIS region reaching its highest intensity value in the UV region. The intensity of the R(ω) spectra is relatively higher along the E||x than the E||z, which supports optical anisotropy. The reflectivity increases from 2% to 30% moving from the IR to UV region, indicating that *p*-SiPN is a highly transparent material for light at lower energy.

The refractive index η(ω) (Figure 5e) is crucial to study the nature of light propagation through the monolayer. Especially the value of η(ω=0) is defined to be the static refractive index, and is found to be 1.37(1.25) for the E||x(z) direction. The η(0) value is slightly smaller than that of *p*-BCN (1.43) and penta-graphene (1.62) [53].

The energy-loss function E(ω) in the energy range of (3.43–10.00) eV is shown in Figure 5f. It is very consistent for both light directions up to 8.16 eV, with a slight enhancement seen above 9.20 eV along the E||x direction compared to that of E||z. However, when compared to pentagraphene [53], the E(ω) intensity as observed in *p*-SiPN is lower, suggesting minimal energy loss, which makes it suitable for energy harvesting.

## 4. Conclusions

In summary, we employed DFT to predict a new penta *p*-SiPN monolayer, and verified its structural, mechanical, dynamical, and thermodynamical stability. The *p*-SiPN is a relatively soft and elastic material with high elastic strength, and it possesses mechanical isotropy. It is a wide and indirect bandgap semiconductor, with tunable bandgap against applied biaxial strain. Additionally, the optical anisotropy and strong long-energy range UV light absorbance, with small reflectivity and energy loss make *p*-SiPN a highly desirable and promising candidate material in nanomechanics and optoelectronics device applications.

## Figures and Tables

**Table 1:** The elastic tensors $C_{ij=1,2,6}$ (N/m), Young’s modulus $Y_{x/y}$ (N/m), and Poisson’s ratio $\nu_{x/y}$ as obtained from this work in comparison to other ternary penta monolayers referenced in the table below.

Materials	Ref	$C_{11}$	$C_{22}$	$C_{12}$	$C_{66}$	$Y_{x}$	$Y_{y}$	$\nu_{x}$	$\nu_{y}$
*p*-SiPN	This work	91.76	92.98	3.29	44.23	91.64	92.86	0.035	0.036
*p*-SiCN	Ref. [24]	132.15	133.59	−17.44	74.80	129.88	131.29	−0.131	−0.132
*p*-BNSi	Ref. [23]	114.46	112.21	12.76	48.97	113	109	0.11	0.11
*p*-BCN	Ref. [45]	210.15	170.77	4.27	102.93	210.05	170.66	0.020	0.025
	Ref. [21]	223.56	189.16	4.90	104.80	223.45	189.03	0.022	0.026
*p*-CNP	Ref. [22]	173.32	183.57	4.52	99.01	172	190	-	-

**Table 2:** The computed Voigt and Reuss shear ($G^{V}$, $G^{R}$) and area ($K^{V}$, $K^{R}$) moduli, and the elastic anisotropic indices ($A^{\mathrm{SU}}$, $A^{\mathrm{Ranganathan}\;}$, $A^{\mathrm{Kube}}$) as obtained from this work in comparison to other ternary penta monolayers referenced in table below.

Materials	Ref	$G^{V}$	$G^{R}$	$K^{V}$	*K* ${}^{R}$	$A^{\mathrm{SU}}$	$A^{\mathrm{Ranganathan}\;}$	$A^{\mathrm{Kube}\;}$
*p*-SiPN	This work	44.38	44.38	47.83	47.82	0.0000	0.0001	0.0000
*p*-BCN	Refs. [45,46]	98.01	97.21	97.36	96.32	0.0116	0.0273	0.0069

**Table 3:** The static refractive index η(0), dielectric constant $\varepsilon_{1}\left(0\right)$, and absorption edge A${}_{e}$(eV) of *p*-SiPN, in comparison to other investigated ternary pentagonal monolayers referenced below, for both E||x and E||z light polarization directions.

MLs	Methods	η(0)		$\varepsilon_{1}\left(0\right)$		$A_{e}$ (eV)	
		E||x	E||z	E||x	E||z	E||x	E||z
*p*-SiPN	GGA	1.37	1.25	1.88	1.55	3.43	3.44
p-SiCN [24]	GGA	3.17	1.30	10.01	1.98	-	-
p-BCN [45]	GGA	1.43	-	2.04	-	1.69	-

## Data Availability

The data will be available upon request.

## Supplementary Materials

The following supporting information can be downloaded at: https://www.mdpi.com/article/10.3390/nano12224068/s1.
