# Supplementary material for: First-Principles Prediction of New 2D p-SiPN: A Wide Bandgap Semiconductor
Source: Nanomaterials (Basel). 2022 Nov 18;12(22):4068. doi: 10.3390/nano12224068 (PMC9698478; doi:10.3390/nano12224068)
Supplement: Supplementary file 1 [file nanomaterials-12-04068-s001.zip › nanomaterials-2037946-supplementary.pdf]

First-principles Prediction of New 2D p-SiPN: A Wide Bandgap Semiconductor

Shambhu Bhandari Sharma[1], I. A. Qattan[1*], Santosh KC[2,] and Sufian Abedrabbo[1]

[1]Department of Physics, Khalifa University of Science and Technology, P.O. Box 127788, Abu Dhabi, United Arab Emirates

[2]Chemical and Materials Engineering, San Jose State University, San Jose, California 95112, USA

E-mail: Corressponding Authors*: issam.qattan@ku.ac.ae

The lattice parameters and coordinates of the optimized geometry for *p*-SiPN in the POSCAR format.

```
Penta-SiPN
   1.0
   4.4108000000     0.0000000000     0.0000000000
   0.0000000000     4.4340130000     0.0000000000
   0.0000000000     0.0000000000    24.129358000
  Si   P   N
   2   2   2
   Direct
0.49155556   1.01580062   0.21341949
0.00847883   0.47854031   0.21383215
0.38103576   0.61818178   0.27263945
0.88122902   0.87622697   0.15460863
0.14474674   0.14526965   0.18389899
0.64461980   0.34909833   0.24334954
```
